# Combination of Neuroprotective and Regenerative Agents for AGE-Induced Retinal Degeneration: In Vitro Study

**DOI:** 10.1155/2017/8604723

**Published:** 2017-05-09

**Authors:** Guzel Bikbova, Toshiyuki Oshitari, Takayuki Baba, Shuichi Yamamoto

**Affiliations:** Department of Ophthalmology and Visual Science, Chiba University Graduate School of Medicine, Inohana 1-8-1, Chuo-ku, Chiba, Chiba Prefecture 260-8670, Japan

## Abstract

To determine the most effective combination of neuroprotective and regenerative agents for cultured retinal neurons from advanced glycation end products- (AGEs-) induced degeneration, retinal explants of 7 adult Sprague-Dawley rats were three-dimensionally cultured in collagen gel and incubated in serum-free media and in 7 media; namely, AGEs, AGEs + 100 *μ*M citicoline, AGEs + 10 ng/mL NT-4, AGEs + 100 *μ*M TUDCA, AGEs + 100 *μ*M citicoline + TUDCA (doublet), and AGEs + 100 *μ*M citicoline + TUDCA + 10 ng/mL NT-4 (triplet) were examined. The number of regenerating neurites was counted after 7 days of culture, followed by performing TUNEL and DAPI staining. The ratio of TUNEL-positive cells to the number of DAPI-stained nuclei was calculated. Immunohistochemical examinations for the active form of caspase-9 and JNK were performed. All of the neuroprotectants increased the number of neurites and decreased the number of TUNEL-positive cells. However, the number of neurites was significantly higher, and the number of TUNEL-positive cells and caspase-9- and JNK-immunopositive cells was fewer in the retinas incubated with the combined three agents. Combination solutions containing citicoline, TUDCA, and NT-4 should be considered for neuroprotective and regenerative therapy for AGE-related retinal degeneration.

## 1. Introduction

The gradual accumulation of glycated proteins, lipids, and nucleic acid is a common process in normal aging, and the accumulation increases the oxidative stress and triggers further protein modifications. These changes impair the defense mechanisms of the organism [1, 2]. Advanced glycation end products (AGEs) are proteins or lipids that become glycated after exposure to sugars, and AGEs are prevalent in the diabetic vasculature. Several studies have demonstrated that there is an extensive relationship between the development of vascular complications with aging such as in cases of kidney failure, diabetes, and accumulation of AGEs in tissues [3]. The use of two AGEs inhibitors has been shown to prevent the changes in the retina, kidney, and neuronal tissues in animal models of diabetes [4–6]. Studies of the receptors of AGE, or RAGE, have shown that high levels of RAGEs and AGEs together with an activation of NF-*κ*B were present in cardiomyocyte of aged rats [7].

The results of our recent study showed that there was an increase in the expression of NF-*κ*B and SP1 in AGEs-exposed cultured rat retinal neurons suggesting that AGEs exposure enhances the expression of the* RAGE* gene [8]. JNK and p38 belong to the mitogen-activated protein kinase (MAPK) family and respond to stress stimuli and cellular apoptosis pathways [9]. Our earlier study found that exposure of cultured retinal tissues to AGEs activated both JNK and p38 at 24 h. However, 7 days after the exposure, only activated JNK was present suggesting its importance in the retinal apoptosis caused by AGEs exposure [10]. Our studies confirmed that glycation may play a role in the pathogenesis of retinal diabetic neuropathy by triggering different mechanisms resulting in neuronal dysfunction.

We have studied the neuroprotective and regenerative effects of different neurotrophic factors, such as citicoline [11], neurotrophin-4 (NT-4) [12], glial cell line-derived neurotrophic factor (GDNF), hepatocyte growth factor (HGF), and tauroursodeoxycholic acid (TUDCA) [8]. NT-4 was found to enhance neurite regeneration in AGEs-exposed retinas more than the other factors, suggesting its potential as an axoprotectant for neuronal diseases associated with high levels of AGEs.

Thus, the purpose of this study was to determine whether a combination of therapeutic agent will have greater neuroprotective abilities on cultured retinal cells exposed to AGEs. To accomplish this, we used a retinal culture system in which the retina was exposed to excessive AGEs and applied individual or combination of neuroprotective agents to the culture media.

## 2. Materials and Methods

### 2.1. Animals

Seven-week-old male Sprague-Dawley (SD) rats (Japan SLC Co., Hamamatsu, Japan) were used. All of the procedures were performed in accordance with the ARVO Statement on the Use of Animals in Ophthalmic and Vision Research. The experimental protocol was approved by the committee on the Ethics of Animal Experiments of Chiba University. The protocol was renewed every year by Chiba University (Permit numbers: Dou 26-157, Dou 25-68, and Dou 24-40).

### 2.2. Three-Dimensional Collagen Gel Culture of Rat Retinal Explants

Seven SD rats were killed by an overdose of ether. The retinas were isolated under sterile conditions and cut into square pieces of 0.16 mm^2^ with a sharp razor blade. The retinal explants were three-dimensionally cultured on collagen gels as described in detail [13–17]. Previous studies have shown that 4 to 480 *μ*g/mL of glycated products was circulating in diabetic patients [2]. In our recent study, 10 *μ*g/mL AGE-bovine serum albumin (BSA) was used as a low-dose AGE medium, but this concentration was high enough to induce neuronal apoptosis in the cultured retinas [18]. Thus, 100 *μ*g/mL AGE-BSA was used as a high-dose AGEs medium.

The retinal explants were incubated in 8 different types of media:

1. serum-free control culture media, N,
2. 100 *μ*g/mL glucose-AGE-BSA (Cyclex Co., Nagano, Japan),
3. glucose-AGE + RAGE inhibitor (R&D Systems, Minneapolis, MN) media,
4. glucose-AGE + 100 *μ*M citicoline (R&D Systems) media,
5. glucose-AGE + 100 *μ*M citicoline + 10 ng/mL NT-4,
6. glucose-AGE + 100 *μ*M TUDCA (WAKO, Osaka, Japan),
7. glucose-AGE + 100 *μ*M citicoline + 100 *μ*M TUDCA (doublet combination),
8. glucose-AGE + 100 *μ*M citicoline + 100 *μ*M TUDCA + 10 ng/mL NT-4 (triplet combination).

The media containing citicoline and TUDCA, media 7, will be referred to as the doublet media, and the media containing citicoline, TUDCA, and NT-4 will be referred to as the triplet media. The explants were maintained at 37°C and exposed to 5% CO_2_.

The serum-free media contained 7.5 mM glucose, 5 *μ*g/mL insulin, 16.1 *μ*g/mL putrescine, 10% bovine serum albumin, 3.7 mg/mL NaHCO_3_, 5.2 mg/L Na_2_SeO_3_, and 3.6 mg/mL HEPES in minimum essential medium as described [8, 12–18]. No additional albumin was used for the control medium except for the addition of glycated BSA because AGE-BSA added only 10% to the total BSA in the culture media.

### 2.3. TUNEL Staining

To determine whether apoptosis had occurred, the retinal explants were fixed in 4% paraformaldehyde after 7 days in culture and sectioned with a cryostat. Then TUNEL staining was carried out with an apoptosis detection kit (Chemicon International, Temecula, CA) according to the manufacturer's instructions. Nonspecific signals were detected by omitting the enzyme reaction. Sections were costained with 4,6-diamidino-2-phenyl indole (DAPI, Polyscience Inc., Warrington, PA). For quantitative analyses, the ratio of the number of TUNEL-positive cells to the total number of DAPI-stained nuclei in the ganglion cell layer (GCL) was determined. A total of 21 sections from the 7 explants/group were studied, and the results were used for the statistical analyses. Total number of nuclei counted in each medium was 187 (N), 295 (AGEs), 288 (NT-4), 234 (citicoline), 262 (TUDCA), 231 (doublet), and 248 (triplet).

### 2.4. Immunohistochemistry

After 7 days in culture, the retinal explants were fixed in paraformaldehyde and cryosections were cut. After blocking the sections in 5% goat serum and 3% bovine serum in 0.1 M phosphate buffer saline, they were incubated with rabbit anti-phosphorylated JNK antibody (p-JNK; 1 : 100, Santa Cruz Biotechnology, Santa Cruz, CA) or the active form of caspase-9 antibody (1 : 100, Cell Signaling Technology Japan, Tokyo, Japan) overnight at 4°C. The sections were then incubated with fluorescein isothiocyanate-conjugated anti-rabbit IgG for one hour. Sections were costained with DAPI to make the nuclei visible. The number of p-JNK- and active form of caspase-9-positive cells in the GCL was counted. For quantitative analyses, the number of immunopositive cells in the GCL was expressed relative to the total number of DAPI-stained nuclei. Twenty-six sections were used from the 7 explants/group, and the total number of nuclei counted was 209 (N), 268 (AGEs), 245 (NT-4), 212 (citicoline), 223 (TUDCA), 198 (doublet), and 223 (triplet) media.

### 2.5. Assessment of Regenerating Neurites

The number of neurites regenerating from the explants was counted under a phase-contrast microscope after 7 days in culture when the number of regenerating neurites is high [12–18]. Branched neurites were counted as one. The number of explants examined was 77 in the control groups including serum-free media (N), N + NT-4, N + citicoline, N + TUDCA, N + doublet, N + triplet, 19 in the glucose-AGE-BSA group, 17 in the glucose-AGE-BSA + NT-4, 6 in the glucose-AGE-BSA + inhibitor, 19 in the glucose-AGE-BSA + citicoline, 21 in the glucose-AGE-BSA + TUDCA, 18 in the glucose-AGE-BSA + doublet, and 18 in the glucose-AGE-BSA + triplet.

Statistical analyses were carried out by one-way analysis of variance with Bonferroni's multiple comparisons tests. A *P* < 0.05 was considered significant.

## 3. Results

### 3.1. Detection of Apoptosis

**Figure 1:**
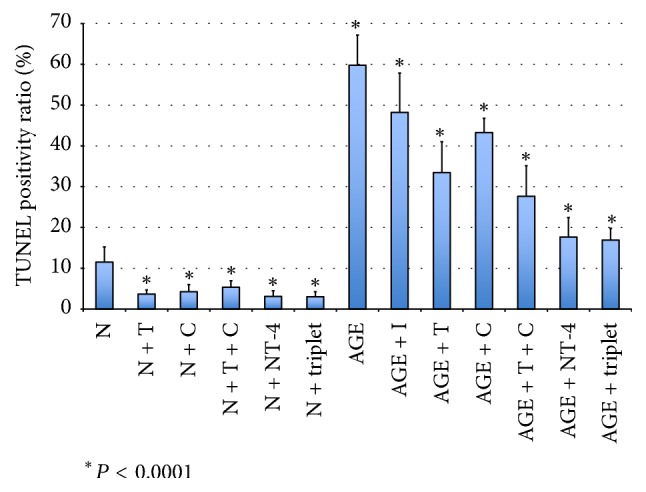
Graph showing the ratio of TUNEL-positive to the total number of retinal neuronal cells in retinal explants in different media. N, serum-free media; AGE, glucose-AGE-BSA media; NT-4, neurotrophin-4 media; C, citicoline media; T, tauroursodeoxycholic acid media; I, RAGE inhibitor.

To determine the effect of AGEs and neurotrophic factors on the retinas in culture, the number of TdT-dUTP nick-end labeling- (TUNEL-) positive cells in the GCL was counted. The majority of the TUNEL-positive cells was detected in the GCL because all of the retinal ganglion cells (RGCs) were axotomized to isolate the retina [8, 12–18]. In retinas cultured in glucose-AGE-BSA, the number of TUNEL-positive cells in the GCL was significantly higher than that in the serum-free control medium (59.8 ± 7.4% versus 11.5 ± 3.7%, *P* < 0.0001; Figure 1). In the retinas incubated with RAGE inhibitor and cultured in AGE-BSA, the number of TUNEL-positives was significantly lower than in glucose-AGE-BSA without the inhibitor (48.2 ± 9.7% versus 59.8 ± 7.4%; *P* = 0.003). In the citicoline supplemented media with or without AGE-BSA, the numbers of TUNEL-positive cells were significantly lower than that in AGE-BSA free media without citicoline (4.2 ± 1.8% versus 11.5 ± 3.7%; *P* = 0.0147) and in glucose-AGE-BSA without citicoline (43.3 ± 3.6% versus 59.8 ± 7.4%; *P* < 0.0001; Figure 1). In the retinas cultured in TUDCA with or without AGE-BSA, the number of TUNEL-positive cells was significantly lower than that in the AGE-BSA free media without TUDCA (3.7 ± 1.0% versus 11.5 ± 3.7%; *P* < 0.0001) and in glucose-AGE-BSA without TUDCA (33.4 ± 7.6% versus 59.8 ± 7.4%; *P* < 0.0001; Figure 1). In the combined citicoline and TUDCA (doublet) exposed retinas, the number of TUNEL-positive cells was significantly lower than in the AGE-BSA free media without the doublet media (5.3 ± 1.6% versus 11.5 ± 3.7%; *P* < 0.0001) and in the glucose-AGE-BSA without the doublet media (37.7 ± 7.5% versus 59.8 ± 7.4%; *P* < 0.0001; Figure 1). In the NT-4 incubated retinas, the number of TUNEL-positive cells was significantly lower than in the AGE-BSA-free media without NT-4 (3.1 ± 1.4% versus 11.5 ± 3.7%; *P* < 0.0001) and in glucose-AGE-BSA without NT-4 (17.6 ± 4.8% versus 59.8 ± 7.4%; *P* < 0.0001; Figure 1). In the retinas incubated in the citicoline, TUDCA, and NT-4 triplet media, the number of TUNEL-positive cells was significantly lower than in the AGE-BSA-free media without the triplet media (3.0 ± 1.2% versus 11.5 ± 3.7%; *P* < 0.0001) and in glucose-AGE-BSA without the triplet media (16.9 ± 2.9% versus 59.8 ± 7.4%; *P* < 0.0001; Figure 1).

TUNEL-positive cells in the inner nuclear layer (INL) and the outer nuclear layer (ONL) were extremely smaller than that in the GCL because all of the RGCs were axotomized to isolate the retina during the procedure and because inner retinas in a collagen gel could be maintained for a long period of culture because of the stable condition. Thus, the results of TUNEL-positive cells in the INL and the ONL should be interpreted with caution (see Supplemental Figures  1 and 2 in the Supplementary Material available online at https://doi.org/10.1155/2017/8604723).

### 3.2. p-JNK Immunopositivity in Ganglion Cell Layer

**Figure 2:**
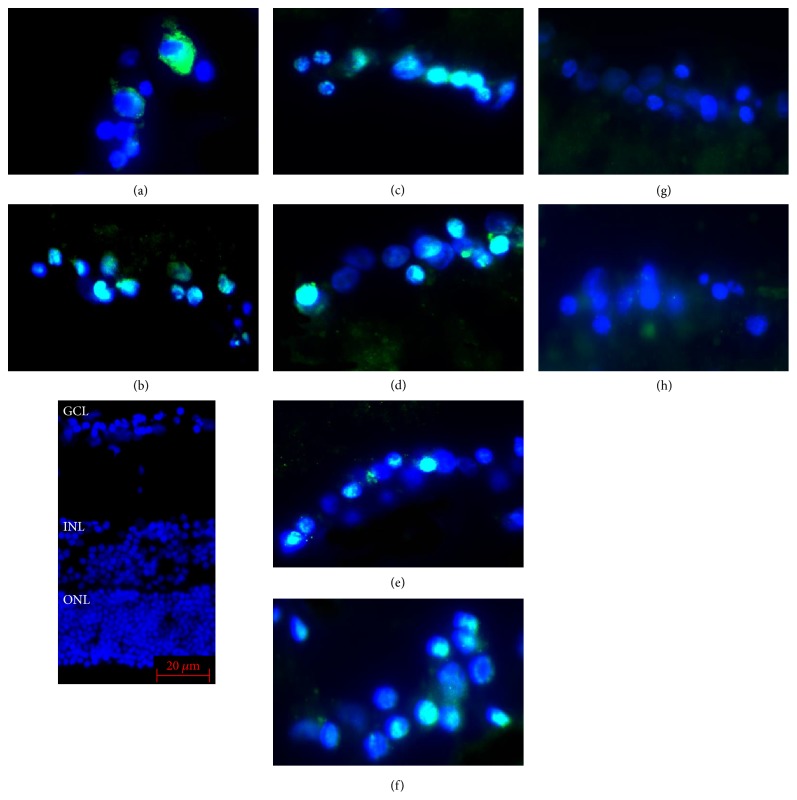
Representative photomicrographs of p-JNK-immunopositive cells in the ganglion cell layer (GCL). The number of immunopositive cells in the GCL is significantly higher than in serum-free control media (a) and in retinas cultured in glucose-AGE-BSA media (b), and in AGEs-exposed retinas supplemented with NT-4 media (glucose-AGE-BSA + NT-4) (g), and in triplet media (glucose-AGE-BSA + citicoline + TUDCA + NT-4) (h), with doublet media (glucose-AGE-BSA + citicoline + TUDCA) (f), with citicoline (glucose-AGE-BSA + citicoline) (d), with TUDCA (glucose-AGE-BSA + TUDCA) (e), and with RAGE inhibitor media (glucose-AGE-BSA + RAGE-I) (c), the number of JNK-immunopositive cells is fewer than that in AGEs-exposed retinas without the neurotrophic factors. The blue staining shows the DAPI-stained nuclei. Bar = 20 *μ*m. GCL: ganglion cell layer, INL: inner nuclear layer, and ONL: outer nuclear layer.

**Figure 3:**
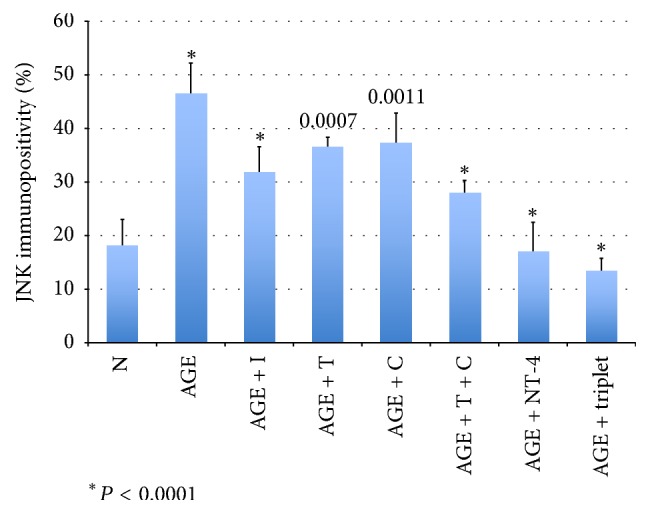
Graph showing ratios of JNK-immunopositive cells to all cells in the GCL. N: serum-free media; AGE: glucose-AGE-BSA; NT-4: neurotrophin-4. C: citicoline; T: tauroursodeoxycholic acid.

The sections were immunostained for p-JNK to determine whether p-JNK was expressed in retinas exposed to AGEs and to determine the effect of neurotrophic factors on this expression. In retinas cultured with glucose-AGE-BSA, the number of p-JNK-immunopositive cells was higher than in serum-free control medium (46.5 ± 5.8% versus 13.9 ± 5.1%; *P* < 0.0001; Figures 2 and 3). The number of immunopositive cells in retinas cultured in glucose-AGE-BSA and incubated with NT-4 was fewer than that in glucose-AGE-BSA without NT-4 (16.9 ± 5.6% versus 46.5 ± 5.8%; *P* < 0.0001; Figures 2 and 3). In retinas cultured in glucose-AGE-BSA supplemented with the triplet media, the number of immunopositive cells was significantly fewer than that in glucose-AGE-BSA without the triplet media (13.9 ± 2.4% versus 46.5 ± 5.8%; *P* < 0.0001; Figures 2 and 3). In retinas cultured in glucose-AGE-BSA supplemented with the doublet media, the number of immunopositive cells was significantly fewer than that in glucose-AGE-BSA without the doublet media (27.8 ± 2.5% versus 46.5 ± 5.8%; *P* < 0.0001). The number of immunopositive cells in retinas cultured in AGE-BSA supplemented with citicoline was significantly fewer than that in glucose-AGE-BSA without citicoline (37.3 ± 5.6% versus 46.5 ± 5.8%; *P* = 0.0011; Figures 2 and 3). The number of immunopositive cells in retinas cultured in AGE-BSA supplemented with TUDCA was significantly fewer than that in glucose-AGE-BSA without TUDCA (36.5 ± 1.9% versus 46.5 ± 5.8%; *P* = 0.0007; Figures 2 and 3). The number of immunopositive cells in retinas cultured in AGE-BSA supplemented with RAGE inhibitor was significantly fewer than that in glucose-AGE-BSA without the RAGE inhibitor (31.8 ± 14.8% versus 46.5 ± 5.8%; *P* < 0.0001; Figures 2 and 3).

### 3.3. Caspase-9 Immunopositivity in Ganglion Cell Layer

**Figure 4:**
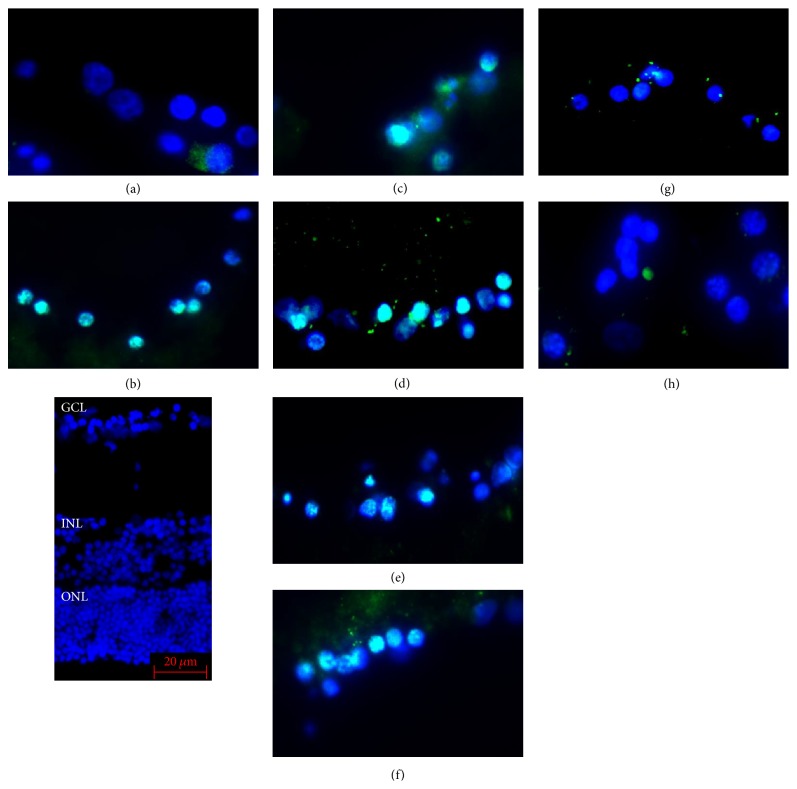
Representative photomicrographs of cultured retinas exposed to AGEs. Immunopositive caspase-9-positive cells in the ganglion cell layer (GCL). In retinas cultured in glucose-AGE-BSA media (b), the number of immunopositive cells in the GCL is significantly higher than in serum-free control media (a). In AGEs-exposed retinas supplemented with NT-4 media (glucose-AGE-BSA + NT-4) (g), in the triplet media (glucose-AGE-BSA + citicoline + TUDCA + NT-4) (h), in the doublet media (glucose-AGE-BSA + citicoline + TUDCA) (f), in citicoline media (glucose-AGE-BSA + citicoline) (d), in TUDCA media (glucose-AGE-BSA + TUDCA) (e), and in RAGE inhibitor media (glucose-AGE-BSA + RAGE-I) (c), the number of caspase-9-immunopositive cells is fewer than that in AGEs-exposed retinas without the neurotrophic factors. The blue staining shows the DAPI-stained nuclei. Bar = 20 *μ*m. GCL: ganglion cell layer, INL: inner nuclear layer, and ONL: outer nuclear layer.

**Figure 5:**
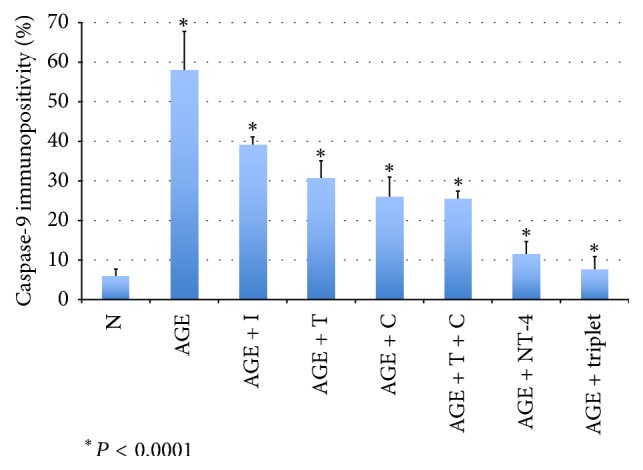
Graph showing ratios of caspase-9-immunopositive cells to all cells in the GCL. N: serum-free media; AGE: glucose-AGE-BSA; NT-4: neurotrophin-4. C: citicoline; T: tauroursodeoxycholic acid.

The sections were immunostained to determine whether caspase-9 was expressed in retinas exposed to AGEs and effect of neurotrophic factors on this expression. In retinas cultured with glucose-AGE-BSA, the number of caspase-9 immunopositive cells was higher than in serum-free control medium (58.0 ± 9.8% versus 5.9 ± 1.9%; *P* < 0.0001; Figures 4 and 5). The number of immunopositive cells in retinas cultured in glucose-AGE-BSA incubated with NT-4 was fewer than that in glucose-AGE-BSA without NT-4 (11.5 ± 3.2% versus 58.0 ± 9.8%; *P* < 0.0001; Figures 4 and 5). In retinas cultured in glucose-AGE-BSA supplemented with the triplet media, the number of caspase-9-positive cells was significantly fewer than that in glucose-AGE-BSA without the triplet media (7.7 ± 3.2% versus 58.0 ± 9.8%; *P* < 0.0001; Figures 4 and 5). In retinas cultured in glucose-AGE-BSA supplemented with the doublet media, the number of caspase-9-positive cells was significantly fewer than that in glucose-AGE-BSA without the doublet media (25.5 ± 1.9% versus 58.0 ± 9.8%; *P* < 0.0001). The number of caspase-9-positive cells in retinas cultured in AGE-BSA supplemented with citicoline was significantly fewer than that in glucose-AGE-BSA without citicoline (25.9 ± 5.1% versus 58.0 ± 9.8%; *P* < 0.0001; Figures 4 and 5). The number of caspase-9-positive cells in retinas cultured in AGE-BSA supplemented with TUDCA was significantly fewer than that in glucose-AGE-BSA without TUDCA (30.8 ± 4.3% versus 58.0 ± 9.8%; *P* < 0.0001; Figures 4 and 5). The number of caspase-9-positive cells of retinas cultured in AGE-BSA supplemented with RAGE inhibitor was significantly fewer than that in glucose-AGE-BSA without the RAGE inhibitor (39.0 ± 2.1% versus 58.0 ± 9.8%; *P* < 0.0001; Figures 4 and 5).

### 3.4. Regenerating Neurites

**Figure 6:**
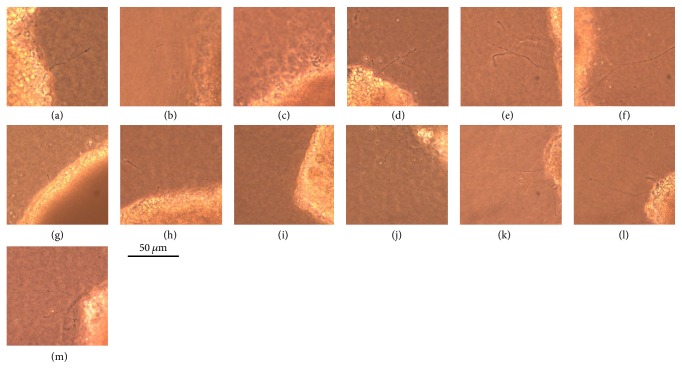
Representative photographs of regenerating neurites from retinal explants. Regenerating neurites are seen under phase-contrast microscope. In the control serum-free media (a) neurites with normal length are present. In retinas cultured in glucose-AGE-BSA (g), the neurites are shorter, and the number of neurites are fewer. In AGEs-exposed retinas supplemented with NT-4, in doublet media and triplet media (glucose-AGE-BSA + NT-4 (l), glucose-AGE-BSA + doublet (j), and glucose-AGE-BSA + triplet (k)), the neurites are longer and thicker than in AGEs-exposed retinas (g). In AGEs-exposed retinas supplemented with citicoline, TUDCA, and RAGE inhibitor (glucose-AGE-BSA + citicoline (h), glucose-AGE-BSA + TUDCA (i), and glucose-AGE-BSA + inhibitor (m)), the neurites are longer and thicker than in AGEs-exposed retinas (g). Control group: serum-free media + citicoline (b), serum-free media + TUDCA (c), serum-free media + doublet (d), serum-free media + triplet (e), and serum-free media + NT-4 (f).

**Figure 7:**
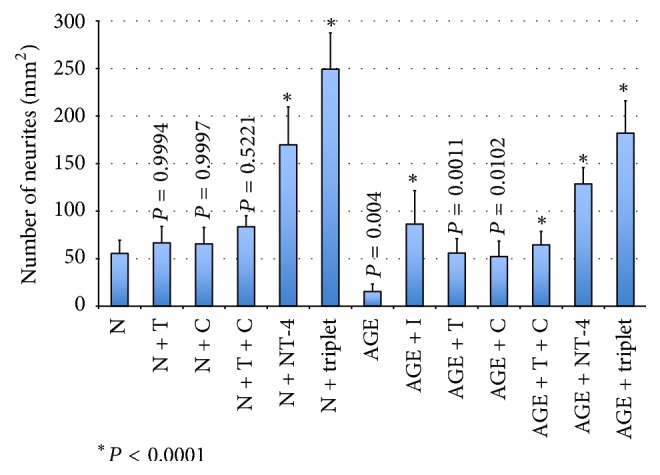
Graph showing the number of regenerating neurites in all groups. N: serum-free media; AGE: glucose-AGE-BSA; NT-4: neurotrophin-4. C: citicoline; T: tauroursodeoxycholic acid.

In retinas incubated with AGEs, the number of regenerating neurites was fewer than in retinas without AGE (15.5 ± 7.9/mm^2^ versus 55.5 ± 14.0/mm^2^; *P* = 0.004; Figures 6 and 7). The retinas incubated in the neurotrophic factors and their combinations, citicoline, TUDCA, NT-4, doublet, and triplet, had more regenerating neurites than that in serum-free media without neurotrophic factors (65.6 ± 17.3/mm^2^ versus 55.5 ± 14.0/mm^2^, *P* = 0.9997; 66.6/mm^2^ ± 17.3/mm^2^ versus 55.5 ± 14.0/mm^2^, *P* = 0.9994; 169.8 ± 40.0/mm^2^ versus 55.5 ± 14.0/mm^2^, *P* < 0.0001; 83.5 ± 11.6 versus 55.5 ± 14.0/mm^2^, *P* = 0.5221; 249.3 ± 38.1/mm^2^ versus 55.5 ± 14.0/mm^2^, *P* < 0.0001) (Figures 6 and 7). However, the differences were statistically different only in the NT-4 and triplet group. Also the agents and their combinations increased the number of regenerating neuritis in AGEs-exposed retinas but the most significant regenerative effect was found in the NT-4 and triplet groups: 128.7 ± 17.1/mm^2^ versus 15.5 ± 7.9/mm^2^, *P* < 0.0001 (NT-4 in AGE-BSA versus without NT-4 in AGE-BSA); 181.9 ± 34.0/mm^2^ versus 15.5 ± 7.9/mm^2^, *P* < 0.0001 (triplet in AGE-BSA versus without triplet in AGE-BSA); 64.6 ± 14.1/mm^2^ versus 15.5 ± 7.9/mm^2^, *P* < 0.0001 (the doublet in AGE-BSA versus without doublet in AGE-BSA); 52.3 ± 16.2/mm^2^ versus 15.5 ± 7.9/mm^2^, *P* = 0.0102 (citicoline in AGE-BSA versus without citicoline in AGE-BSA); 56.0 ± 15.2/mm^2^ versus 15.5 ± 7.9/mm^2^, *P* = 0.0011 (TUDCA in AGE-BSA versus without TUDCA in AGE-BSA), and 86.5 ± 35.2/mm^2^ versus 15.5 ± 7.9/mm^2^, *P* < 0.0001 (RAGE inhibitor supplemented in AGE-BSA incubated retinas versus without RAGE inhibitor in AGE-BSA; Figures 6 and 7). In AGEs-exposed retinas incubated in the triplet media, the numbers of neurites were significantly higher than in the AGEs-exposed retina incubated with NT-4, RAGE inhibitor, citicoline, TUDCA, or the doublet media (*P* < 0.0001).

## 4. Discussion

Citicoline (cytidine 5′-diphosphocholine) is naturally occurring, and it is composed of ribose, cytosine, pyrophosphate, and choline, and it is an intermediate in the synthesis of membrane phospholipids such as phosphatidylcholine [19, 20]. The synthesis of the phospholipids in the central nervous system (CNS) can be controlled by altering of the citicoline concentration [21, 22]. Citicoline has been investigated as a possible therapeutic agent for brain ischemia, Alzheimer's disease (AD), and Parkinson's disease, amblyopia, non-arteritic ischaemic optic neuropathy, and glaucoma [23–26]. It was suggested that the possible mechanism of citicoline's neuroprotective effects is the prevention of the activation of phospholipase A_2_ (PLA_2_), the predominant isoform in membrane and mitochondria, which were shown in a stroke model [27]. It was found that citicoline attenuated the increase in PLA_2_ activity in both the membrane and mitochondrial fractions. Oshitari et al. studied the effect of citicoline on damaged retinal neurons and found an antiapoptotic effect of citicoline in the mitochondria-dependent cell death mechanism and its ability to support axon regeneration. It was chosen as a compound for our combined therapeutic agent because it acted as a mitochondria stabilizer and was participating in neuroprotection.

The results of recent studies have indicated that citicoline eye drops can enhance the visual function of patients with glaucoma without adverse side effects [28, 29]. These results indicated the possible use for citicoline eye drops in patients. Another recent study used citicoline topically to determine if it can protect neurons in a mouse model of diabetic retinopathy [30]. The retinal nerve fiber layer thickness and ganglion cell complex obtained from optical coherence tomography were compared in eyes with and without citicoline treatment. Unfortunately, they could not find any significant difference between the RNFL thickness in the treated and not treated eyes. These results suggest that the use of only one neuroprotective agent may not be sufficient.

TUDCA is known to modulate the endoplasmic reticulum (ER) function to protect cells against ER stress-induced apoptosis [31]. It was selected for our study because of its protective effect on damaged retinal neurons under diabetic stress as an anti-ER reagent [12, 32]. The results of our earlier study indicated that the neuroprotective and regenerative effects of TUDCA were correlated with the suppression of the expression of p38 and p-JNK expression. TUDCA has also been shown to modulate the ER function to protect cells against ER stress-induced apoptosis [12, 32]. ER stress is a characteristic component in various neurodegenerative disorders leading to proapoptotic molecule induction such as the growth arrest of the DNA damage-inducible gene [33, 34]. Considering these features, TUDCA was selected for its protective effect on damaged retinal neurons under diabetic stress as an anti-ER reagent.

In this study, both TUDCA and citicoline significantly reduced not only JNK but also caspase-9 immunopositive cells in the GCL. JNK is known to be activated under ER stress via IRE1*α* [17] and caspase-9 is activated under mitochondria-dependent cell death pathway [15]. Because there is a molecular crosstalk between ER stress related cell death pathway and mitochondria-dependent cell death pathway in this system [12, 17], both TUDCA and citicoline may be able to reduce both JNK and caspase-9 immunopositivities in cultured retinas. Further studies using other systems are needed to elucidate the precise mechanisms of neuroprotection and regeneration with mixed neuroprotectants.

Earlier, we investigated the neuroprotective and regenerative effects of NT-4, which was found to promote the survival and the regeneration of retinal cells incubated in high glucose media. The neuroprotective and regenerative effects of NT-4 were correlated with the reduction in the activation of caspase-9 and caspase-3 [13, 18], PKR-like ER kinase, and C/EBP homologous protein expressions [12], and c-Jun and JNK and p38 expression [32]. In the earlier studies, a concentration 100 ng/mL of NT-4 was used [8]. Considering the administration by eye drops, the concentration 10 ng/mL for combined therapeutic agent was chosen for this study. Even low concentrations of NT-4 were found to significantly increase the rates of survival and regenerating cells. The results of a recent pilot study indicated that topical administration of a total dose of 1 mg nerve growth factor (NGF) per patient with retinitis pigmentosa partly improved the visual function in some of the patients [35]. NT-4 belongs to the NGF family. Thus, NT-4 topical administration may be feasible to treat diabetic patients. Further in vivo studies are required to examine the effect of topical application of NT-4 on neuronal abnormalities in diabetic retinopathy.

NT-4 alone or in combination with doublet (without NT-4) and triplet (with NT-4) significantly increased the number of regenerated neurites and decreased the number of TUNEL-positive cells, which is correlated with decrease in the expressions of caspase-9 and JNK. Thus, combining NT-4, citicoline, and TUDCA provided the maximal neuroprotective and regenerative effect by influencing different pathological pathways which are triggered in diseases associated with AGEs accumulation including diabetic retinopathy.

Chronic exposure of NT-4 may induce trkB receptor downregulation. Thus, we examined the neuroprotective effect of the doublet (without NT-4). Although regenerative effect of the doublet was weak, the doublet media had significant neuroprotective effects on AGEs-exposed retinas. To prevent trkB downregulation, alternate therapies with doublets and triplets in in vivo animal models may be one of the options for long-lasting topical administration of axoprotectants for the treatment of chronic retinal diseases such as diabetic retinopathy.

## 5. Conclusion

A solution containing three neuroprotective and neuroregenerative agents was best in enhancing neurite regeneration in AGEs-exposed retinas more than citicoline, TUDCA, and NT-4 alone. Thus they should be considered as possible agents for neuroprotective and regenerative therapy for diabetic retinopathy. Further in vivo studies will be performed for determining the effects of combination therapies with eye drops in diabetic animal models.

## Supplementary Material


**Supplementary Figure 1. TUNEL-positive cells in the INL**. Total numbers of TUNEL-positive cells in the INL were too small and the data was expressed the number per explant. In the control, the number of TUNEL-positive cells/explant was 4.0±3.6/explant. In retinas supplemented with citicoline, TUDCA and NT-4, the numbers were significantly reduced compared to control (2.3±2.8 vs. 4.0±3.6/explant, 2.5±2.6 vs. 4.0±3.6/explant, 1.3±2.4 vs. 4.0±3.6/explant; *P*<0.05, respectively). In retinas supplemented with doublet and triplet, the numbers were significantly reduced compare to control (0.8±1.1 vs. 4.0±3.6/explant, 0.7±1.2 vs. 4.0±3.6/explant; *P*<0.05, respectively). In AGE-exposed retinas, the number was significantly increased compared to control (11.2±7.2 vs. 4.0±3.6/explant; *P*<0.01). In AGE-exposed retinas incubated with RAGE inhibitor, citicoline, TUDCA, NT-4, doublet and triplet, the numbers were significantly smaller than AGE-exposed retinas without neurotrophic factors (8.0±6.7 vs. 11.2±7.2/explant, 6.0±4.8 vs. 11.2±7.2/explant, 7.7±6.8 vs. 11.2±7.2/explant, 2.5±2.3 vs. 11.2±7.2/explant, 6.0±5.6 vs. 11.2±7.2/explant, 2.8±2.8 vs. 11.2±7.2/explant; *P*<0.05, respectively).
**Supplementary Figure 2. TUNEL-positive cells in the ONL**. Total numbers of TUNEL-positive cells in the ONL were too small and the data was expressed the number per explant. In the control, the number of TUNEL-positive cells/explant was 1.3±1.4/explant. In retinas supplemented with citicoline, TUDCA and NT-4, the numbers were significantly reduced compared to control (0.8±1.1 vs. 1.3±1.4/explant, 1.0±1.2 vs. 1.3±1.4/explant, 0.2±0.5 vs. 1.3±1.4/explant; *P*<0.05, respectively). In retinas supplemented with doublet and triplet, the numbers were significantly reduced compare to control (0.4±0.7 vs. 1.3±1.4/explant, 0.1±0.3 vs. 1.3±1.4/explant; *P*<0.05, respectively). In AGE-exposed retinas, the number was significantly increased compared to control (4.3±3.0 vs. 1.3±1.4/explant; *P*<0.01). In AGE-exposed retinas incubated with RAGE inhibitor, citicoline, TUDCA, and triplet, the numbers were significantly smaller than AGE-exposed retinas without neurotrophic factors (3.9±1.9 vs. 4.3±3.0/explant, 3.2±2.1 vs. 4.3±3.0/explant, 2.4±2.5 vs. 4.3±3.0/explant, 1.0±1.3 vs. 4.3±3.0/explant; *P*<0.05, respectively). In AGE-exposed retinas incubated with NT-4 and doublet, the numbers did not reach statistical significance compared to AGE-exposed retinas without neurotrophic factors (1.1±1.2 vs. 4.3±3.0; *P*=0.059, 2.9±3.3 vs. 4.3±3.0; *P*=0.076, respectively).
