# Supplementary material for: Combination of Neuroprotective and Regenerative Agents for AGE-Induced Retinal Degeneration: In Vitro Study
Source: Biomed Res Int. 2017 May 9;2017:8604723. doi: 10.1155/2017/8604723 (PMC5440790; doi:10.1155/2017/8604723)

Supplemental Figures

Figure 1. **TUNEL-positive cells in the INL**. Total numbers of TUNEL-positive cells in the INL were too small and the data was expressed the number per explant. In the control, the number of TUNEL-positive cells/explant was 4.0±3.6/explant. In retinas supplemented with citicoline, TUDCA and NT-4, the numbers were significantly reduced compared to control (2.3±2.8 vs. 4.0±3.6/explant, 2.5±2.6 vs. 4.0±3.6/explant, 1.3±2.4 vs. 4.0±3.6/explant; $P<0.05$, respectively). In retinas supplemented with doublet and triplet, the numbers were significantly reduced compare to control (0.8±1.1 vs. 4.0±3.6/explant, 0.7±1.2 vs. 4.0±3.6/explant; $P<0.05$, respectively). In AGE-exposed retinas, the number was significantly increased compared to control (11.2±7.2 vs. 4.0±3.6/explant; $P<0.01$). In AGE-exposed retinas incubated with RAGE inhibitor, citicoline, TUDCA, NT-4, doublet and triplet, the numbers were significantly smaller than AGE-exposed retinas without neurotrophic factors (8.0±6.7 vs. 11.2±7.2/explant, 6.0±4.8 vs. 11.2±7.2/explant, 7.7±6.8 vs. 11.2±7.2/explant, 2.5±2.3 vs. 11.2±7.2/explant, 6.0±5.6 vs. 11.2±7.2/explant, 2.8±2.8 vs. 11.2±7.2/explant; $P<0.05$, respectively).

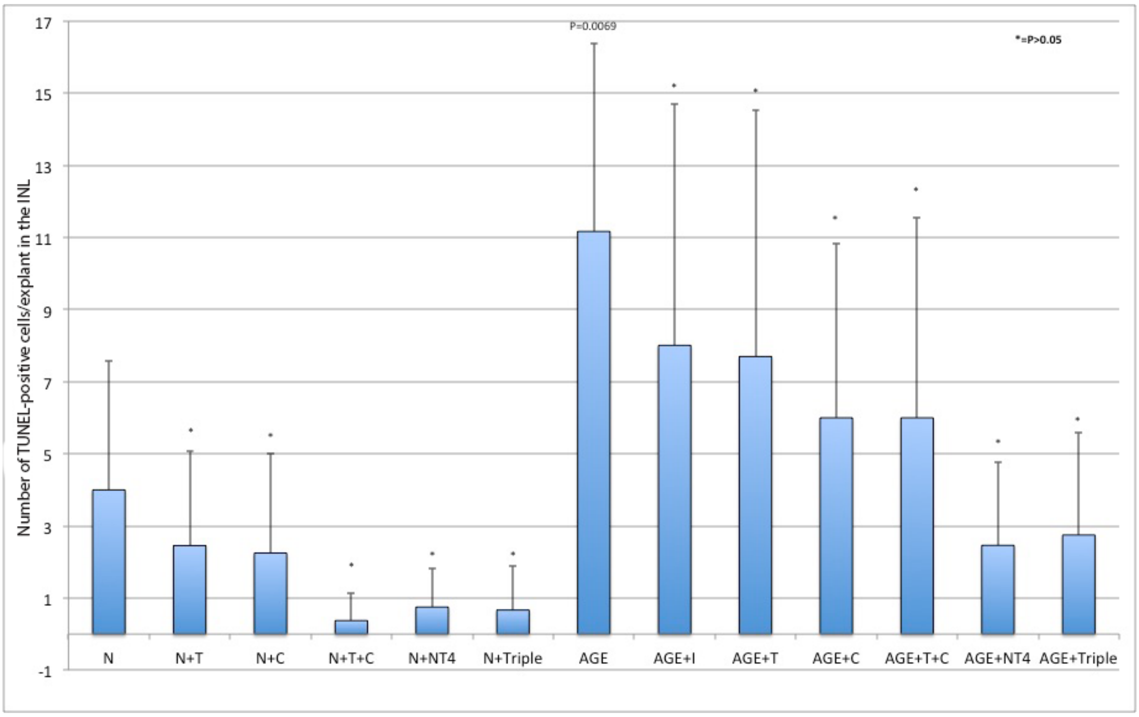

Figure 2. TUNEL-positive cells in the ONL. Total numbers of TUNEL-positive cells in the ONL were too small and the data was expressed the number per explant. In the control, the number of TUNEL-positive cells/explant was 1.3±1.4/explant. In retinas supplemented with citicoline, TUDCA and NT-4, the numbers were significantly reduced compared to control (0.8±1.1 vs. 1.3±1.4/explant, 1.0±1.2 vs. 1.3±1.4/explant, 0.2±0.5 vs. 1.3±1.4/explant; $P<0.05$, respectively). In retinas supplemented with doublet and triplet, the numbers were significantly reduced compare to control (0.4±0.7 vs. 1.3±1.4/explant, 0.1±0.3 vs. 1.3±1.4/explant; $P<0.05$, respectively). In AGE-exposed retinas, the number was significantly increased compared to control (4.3±3.0 vs. 1.3±1.4/explant; $P<0.01$). In AGE-exposed retinas incubated with RAGE inhibitor, citicoline, TUDCA, and

triplet, the numbers were significantly smaller than AGE-exposed retinas without neurotrophic factors (3.9±1.9 vs. 4.3±3.0/explant, 3.2±2.1 vs. 4.3±3.0/explant, 2.4±2.5 vs. 4.3±3.0/explant, 1.0±1.3 vs. 4.3±3.0/explant; $P<0.05$, respectively). In AGE-exposed retinas incubated with NT-4 and doublet, the numbers did not reach statistical significance compared to AGE-exposed retinas without neurotrophic factors (1.1±1.2 vs. 4.3±3.0; $P=0.059$, 2.9±3.3 vs. 4.3±3.0; $P=0.076$, respectively).

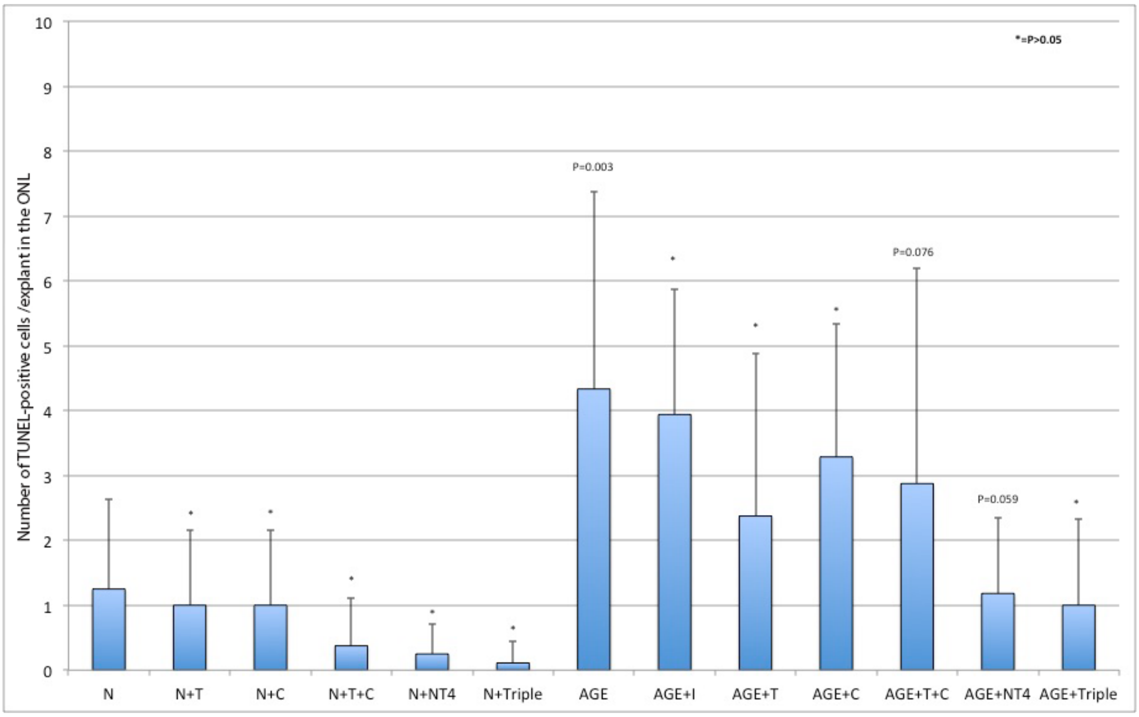

Supplement: Supplementary file 1 — Supplementary Figure 1. TUNEL-positive cells in the INL. Total numbers of TUNEL-positive cells in the INL were too small and the data was expressed the number per explant. In the control, the number of TUNEL-positive cells/explant was 4.0±3.6/explant. In retinas supplemented with citicoline, TUDCA and NT-4, the numbers were significantly reduced compared to control (2.3±2.8 vs. 4.0±3.6/explant, 2.5±2.6 vs. 4.0±3.6/explant, 1.3±2.4 vs. 4.0±3.6/explant; P<0.05, respectively). In retinas supplemented with doublet and triplet, the numbers were significantly reduced compare to control (0.8±1.1 vs. 4.0±3.6/explant, 0.7±1.2 vs. 4.0±3.6/explant; P<0.05, respectively). In AGE-exposed retinas, the number was significantly increased compared to control (11.2±7.2 vs. 4.0±3.6/explant; P<0.01). In AGE-exposed retinas incubated with RAGE inhibitor, citicoline, TUDCA, NT-4, doublet and triplet, the numbers were significantly smaller than AGE-exposed retinas without neurotrophic factors (8.0±6.7 vs. 11.2±7.2/explant, 6.0±4.8 vs. 11.2±7.2/explant, 7.7±6.8 vs. 11.2±7.2/explant, 2.5±2.3 vs. 11.2±7.2/explant, 6.0±5.6 vs. 11.2±7.2/explant, 2.8±2.8 vs. 11.2±7.2/explant; P<0.05, respectively). Supplementary Figure 2. TUNEL-positive cells in the ONL. Total numbers of TUNEL-positive cells in the ONL were too small and the data was expressed the number per explant. In the control, the number of TUNEL-positive cells/explant was 1.3±1.4/explant. In retinas supplemented with citicoline, TUDCA and NT-4, the numbers were significantly reduced compared to control (0.8±1.1 vs. 1.3±1.4/explant, 1.0±1.2 vs. 1.3±1.4/explant, 0.2±0.5 vs. 1.3±1.4/explant; P<0.05, respectively). In retinas supplemented with doublet and triplet, the numbers were significantly reduced compare to control (0.4±0.7 vs. 1.3±1.4/explant, 0.1±0.3 vs. 1.3±1.4/explant; P<0.05, respectively). In AGE-exposed retinas, the number was significantly increased compared to control (4.3±3.0 vs. 1.3±1.4/explant; P<0.01). In AGE-exposed retin [file 8604723.f1.pdf]
